# P-828. Risk of Invasive *Escherichia Coli* (*E. coli)* Disease After Elective Urologic Procedures among Older Adults in the United States

**DOI:** 10.1093/ofid/ofae631.1020

**Published:** 2025-01-29

**Authors:** Maureen P Neary, Maryaline Catillon, Nina Ahmad, Marjolaine Gauthier-Loiselle, Jeroen Geurtsen, Alice Qu, Corinne Willame, Martin Cloutier, Antoine El Khoury, Elie Saade

**Affiliations:** Janssen Global Services, LLC, Raritan, New Jersey; Analysis Group, Inc., New York, New York; Janssen Global Services LLC, Titusville, New Jersey; Analysis Group, Montreal, Quebec, Canada; Janssen Vaccines & Prevention BV, Leiden, Netherlands; Analysis Group, Montreal, Quebec, Canada; Janssen Pharmaceutica NV, Beerse, Antwerpen, Belgium; Analysis Group, Montreal, Quebec, Canada; Janssen Global Services LLC, Titusville, New Jersey; University Hospitals of Cleveland, Cleveland, Ohio

## Abstract

**Background:**

Little is known about the risk of invasive *E. coli* disease (IED) after elective urologic procedures with or without antibiotic prophylaxis. This study aimed to estimate the risk of IED after selected urologic procedures in patients with or without antibiotic prophylaxis, and in controls who had no urologic nor other procedures.

**Table 1.**
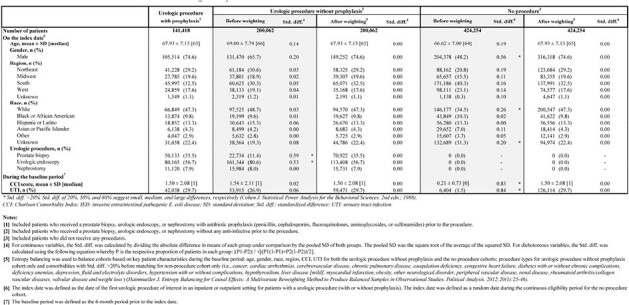
Patient demographic and clinical characteristics

**Methods:**

The Komodo Research Database (01/01/2021–06/30/2023) was used to identify patients ≥60 years old with prostate biopsy, urologic endoscopy, or nephrostomy (index: first urologic procedure date), and a 1:3 control without procedures (index: random date). Patients with urologic procedures were classified into 2 study cohorts based on antibiotic prophylaxis use within 14 days prior to and on index date. Entropy balancing was used to adjust for differences in demographic and clinical characteristics in 6 months pre-index (baseline). IED rates within 30 days post-index were assessed based on recorded diagnosis of *E. coli* sepsis (ICD-10-CM code A41.51); odds ratios (OR) were estimated using weighted logistic regression. Sensitivity analyses within 90 days and using broader claims-based algorithm for IED were conducted.

**Figure 1.**
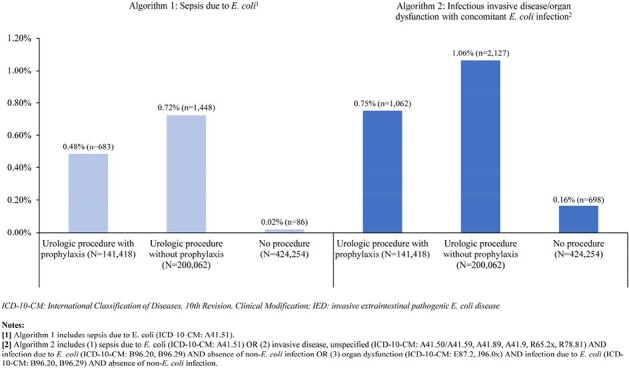
IED rates 30 days post index by IED case-identification algorithm

**Results:**

Overall, 141,418 patients had urologic procedures with antibiotic prophylaxis, 200,062 had them without antibiotic prophylaxis, while control cohort included 424,254 patients who did not have any procedures (Table 1). Within 30 days post-index, IED rates were 0.48% and 0.72% among patients with or without antibiotic prophylaxis, respectively, vs 0.02% among those without procedures (Figure 1). Among patients with urologic procedures, >70% of IED events occurred within 30 days post-index. Patients who had urologic procedures without prophylaxis had a higher risk of IED than those with prophylaxis [OR=1.50, 95% CI (1.37,1.65); Table 2]. Patients without procedures had a lower risk of IED than those with procedures who received antibiotic prophylaxis [OR=0.04, 95% CI (0.03,0.05); Table 2]. Similar results were obtained within 90 days and/or using a broader definition of IED.

**Table 2.**
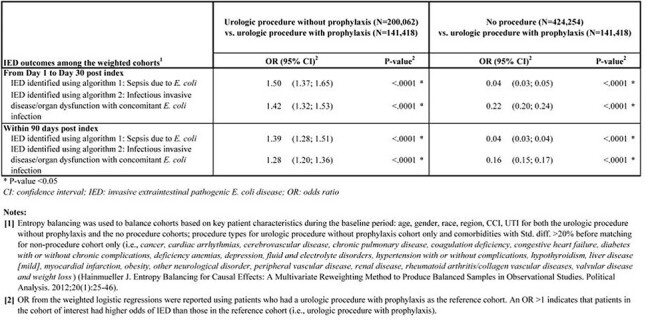
Comparison of IED outcomes among the weighted cohorts using logistic regression analysis

**Conclusion:**

Urologic procedures were associated with an increased risk of IED even when antibiotic prophylaxis was used. Results suggest an unmet need for additional preventative measures to avoid the substantial burden of IED after urologic procedures.

**Disclosures:**

**Maureen P. Neary, PhD, MS**, Janssen Global Services, LLC: Employee of Janssen Global Services, LLC **Maryaline Catillon, PhD**, Janssen Global Services, LLC: Employee of Analysis Group which has received consultancy fees from Janssen Global Services, LLC for the conduct of this study **Nina Ahmad, MD**, Janssen Global Services, LLC: Employee of Janssen Global Services, LLC **Marjolaine Gauthier-Loiselle, PhD**, Janssen Global Services, LLC: Employee of Analysis Group which has received consultancy fees from Janssen Global Services, LLC for the conduct of this study **Jeroen Geurtsen, PhD**, Janssen Vaccines & Prevention BV: Advisor/Consultant|Janssen Vaccines & Prevention BV: Employee of Janssen Vaccines & Prevention BV **Alice Qu, BA**, Janssen Global Services, LLC: Employee of Analysis Group which has received consultancy fees from Janssen Global Services, LLC for the conduct of this study **Corinne Willame, PhD, MPH**, Johnson & Johnson Innovative Medicine: Employee of Johnson & Johnson Innovative Medicine **Martin Cloutier, MSc**, Janssen Global Services, LLC: Employee of Analysis Group which has received consultancy fees from Janssen Global Services, LLC for the conduct of this study **Antoine El Khoury, PhD**, Janssen Global Services: Advisor/Consultant|Janssen Global Services, LLC: Employee of Janssen Global Services, LLC **Elie Saade, MD**, Janssen: Grant/Research Support|Pfizer: Grant/Research Support|Pfizer: advisory board|Sanofi Pasteur: Grant/Research Support|Sanofi Pasteur: speaking and lecture fees and travel reimbursement|Seqirus: Grant/Research Support

